# Blinking kinematics characterization during digital displays use

**DOI:** 10.1007/s00417-021-05490-9

**Published:** 2021-11-15

**Authors:** Cristian Talens-Estarelles, José Juan Esteve-Taboada, Vicent Sanchis-Jurado, Álvaro M Pons, Santiago García-Lázaro

**Affiliations:** Department of Optics & Optometry & Vision Sciences, University of Valencia, Dr. Moliner, 50, 46100 Burjassot, Valencia Spain

**Keywords:** Blink characterization, Blinking kinematics, Digital displays, Spontaneous blinking

## Abstract

**Purpose:**

This study aimed to assess the differences in blinking kinematics while reading on different digital displays and a control condition.

**Methods:**

Thirty-two young healthy individuals were included in this prospective clinical study. The blinks of subjects were recorded for 150 s while reading on a laptop computer, tablet, e-reader, and smartphone and a control condition. Blinks were recorded using an eye-tracking device and were analyzed by means of image analysis to obtain a non-invasive detailed description of the blink movement.

**Results:**

Blink rate decreased when reading on all displays compared to the control (*p* < 0.0005), although no differences were obtained amongst displays (*p* > 0.05). The percentage of incomplete blinks was higher with the computer compared to the control (*p* = 0.043), and lower with the smartphone compared to the rest of the conditions (*p* ≤ 0.015). Blink amplitude was smaller when reading from handheld devices compared to the control (*p* < 0.0005) and the computer (*p* ≤ 0.048). Closing and opening blink durations remained unvaried amongst conditions (*p* > 0.05), while opening and closing speeds were greater for the control and the computer compared to the handheld displays (*p* < 0.0005). Finally, contact and total blink durations were shorter during computer reading compared to the control (*p* = 0.004 and *p* = 0.017, respectively).

**Conclusion:**

Blinking kinematics vary considerably amongst displays and with respect to baseline, with these differences being probably attributed to differences in the way the displays are set up and the cognitive demand of the task. 
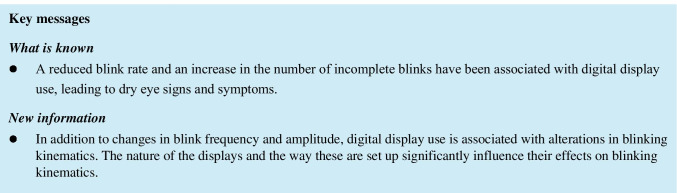

## Introduction

The use of digital displays has increased dramatically over the last decade. In 2007, 55% of households in the European Union had access to the internet [1]. Now the share of households with internet connection has reached the 90% milestone [1]. Nowadays, new forms of digital displays other than desktop computers, such as laptops, tablets, smartphones, or even e-readers have emerged. In this regard, up to 75% of individuals claim to use handheld devices to access the internet daily [1].

According to the American Optometric Association, computer vision syndrome (CVS) “describes a group of eye and vision-related problems that result from prolonged computer, tablet, e-reader and cell phone use” [2]. Previous research points to alterations in the pattern of blinking as one of the main CVS-inducing mechanisms [3–5]. A reduced blink rate and a reduced blink amplitude have both been reported during computer use [3–10]. Given that appropriate blinking is crucial for maintaining ocular surface integrity and tear film stability [11], it is not surprising that digital display use has been listed as a consistent risk factor for dry eye disease (DED) [12, 13]. When it comes to handheld devices, however, research is still limited, and results are conflicting. Nevertheless, conditions such as viewing distance, angle of gaze, and screen size have all been shown to influence blink frequency to different extents [14, 15]. Therefore, one may expect that the differences in the nature of the displays and the ways that they are set up and used may contribute to differences in the blinking pattern and their induced symptoms.

Blinks occur after a complex and coordinated interaction of different skeletal muscles acting antagonistically, with each stage depending on different muscle actions and interactions [16]. Despite its complexity, blinking during digital display use has traditionally been described in terms of blink rate and number of incomplete blinks, this description being, by itself, scant for fully characterizing the process of blinking. Recently, high-speed video cameras, implemented with image processing algorithms, have been used to precisely and non-invasively gather and analyze blink kinematic variables in natural viewing conditions, allowing a full and in-depth description of the process of blinking [17–19].

Consequently, the present study aimed to analyze and compare in detail the kinematic characteristics of blinking while reading on a laptop computer, a tablet, an e-reader, and a smartphone under natural viewing conditions, and after a baseline measurement, implementing a newly developed technique for the non-invasive characterization of blinking. To the authors’ knowledge, this is the first time that the kinematic characteristics of blinking have been assessed and compared while reading on digital displays.

## Methods

### Subjects

Thirty-two young healthy volunteers (12 men and 20 women) participated in the present study. Inclusion criteria were monocular- and binocular-corrected distance and near visual acuity greater than or equal to 20/20, normal binocularity, and normal color vision. Exclusion criteria were prior ocular history of injury, anterior or posterior segment pathology, surgery or current use of topical medications, and contact lens wear or use of artificial tear substitutes. Likewise, participants had no known neurological disorders or took any medications that could affect blinking. To comply with the inclusion/exclusion criteria, subjects with DED were excluded following the guidelines of the TFOS DEWS II diagnostic approach [20].

The study followed the tenets of the Declaration of Helsinki and the permission of the ethical committee of the University of Valencia was obtained. All the subjects were informed about the nature of the study and gave their consent.

### Experimental design

Blinking was assessed in baseline conditions and during a reading task with a laptop computer, a tablet, an e-reader, and a smartphone. For the control condition, subjects were instructed to direct their gaze to a Maltese cross, arranged at eye level and placed 3 m in front of them. For the digital display tasks, participants were instructed to read text shown on the screen of the displays.

Text presented on all 4 displays was matched in font style (Georgia font with black letters on white background), angular size (appropriately chosen for each device for a 0.15 logMAR visual acuity), angular line spacing, number of words per line and page, page angular width (appropriately chosen for each device for a 25° width), and text alignment (left-justified). Also, screen luminance was made equal by adjusting the brightness level setting. With respect to the e-reader, this device is designed to simulate printed paper by reflecting rather than emitting light from behind the screen.

Moreover, digital displays were placed according to typical viewing distance and angle of usage: 60-cm distance and 10° angle below the subject’s eye level for the laptop computer; 45 cm and 25° for the tablet and the e-reader; 30 cm and 45° for the smartphone [21, 22]. Additionally, the 4 screens were set at an inclination angle of 100° from the plane of the desk. An adjustable stand was used to arrange the handheld devices accordingly.

Subjects carried out the tasks with their heads fixed in a chin and forehead rest. To ensure subject comfort and correct alignment with the display screen, the height of the chin rest could be adjusted, as well as that of the chair. The whole experiment was carried out under constant artificial illumination. Room illuminance was maintained at approximately 220 lux on the plane of the subject’s eyes and was provided by indirect lighting to avoid any glare sources. Chroma Meter CL-200 lux meter (Konica Minolta; Ramsey, NJ) was used to measure photometric values. Room temperature and humidity were monitored and remained stable at 23.8 ± 1.6 °C and 44.3 ± 4.5%, respectively.

### Apparatus

During task performance, subjects’ eye movements were recorded with an infrared video–based eye tracker (Cambridge Research Systems Ltd) working at a sample rate of 250 frames per second (fps), without subjects being aware. The High-Speed Visual Eye Tracker (HSVET) consists of a high-speed infrared camera, with a resolution of 320 per 240 pixels, a visible/infrared dichroic beamsplitter, and a chin rest. Due to the configuration of the device, the image of the eye is reflected on the infrared mirror without interfering with the observer’s line of sight, simulating natural viewing conditions (Figure 1(a) in Sanchis-Jurado et al. 2020) [17].

Text material was a book with a recompilation of Allan Poe’s full stories. The text was displayed using Kindle® (2021) reading application (app) (Amazon Inc., Seattle, WA). Text characteristics were matched for all displays and selected from the Kindle app interface. An optical microscope focused on the screens of the devices was used to select text size and line spacing after the trigonometric calculation based on the linear size.

Digital displays included a MacBook Pro laptop computer (Apple Inc., Cupertino, CA) with a 13-inch screen, a resolution of 227 pixels per inch (ppi), a refresh rate of 60 Hz, and a contrast ratio of 1350:1; a third-generation iPad tablet (Apple Inc.) with a 9.7-inch screen, 264 ppi, 60-Hz refresh rate, and 1000:1 contrast ratio; a third-generation Kindle Paperwhite e-reader (Amazon Inc.) based on electronic ink (E-ink) technology, with a 6-inch screen, 330 ppi, and 15:1 contrast ratio (backlight mode turned off); and an iPhone 6 smartphone (Apple Inc.) with a 4.7-inch screen, 326 ppi, 60-Hz refresh rate, and 1000:1 contrast ratio. Digital displays with similar screen characteristics were considered, except for the e-reader, based on E-ink technology, which seeks to simulate printed paper.

### Protocol

All the measurements were taken in the same laboratory. Each condition was tested in separate sessions and with a rest period of 7 days between sessions. Participants completed each of the 5 experimental conditions in the following order: (1) control, (2) computer, (3) tablet, (4) e-reader, and (5) smartphone. The approximate duration of each session was 35 min. To avoid any day-to-day variability, each session was carried out on the same day of the week, at the same time of the day (first thing in the morning, at 9 am), and under constant environmental conditions (temperature and humidity). Additionally, subjects were asked not to use other digital displays before the session and not to drink any beverage containing caffeine 24 h before the measurements. Inter-day reliability was assessed in 8 subjects before initiating the study. Preliminary assessment of day-to-day variability revealed no significant variations in study variables between sessions under the mentioned experimental conditions.

Fifteen minutes before the subject’s visit, the laboratory was acclimatized, and the experimental conditions were set up. Once the subject arrived, he/she received instructions on the session’s task. In the case of reading on a digital display, the subject was given a few minutes to choose between one of the stories from the book and was taught how to handle the device for the reading. To minimize the effects of outdoor conditions on the way to the laboratory, a 15-min acclimatization period was left between the subject’s entry into the room and the start of the task. Then, subjects were seated comfortably and instructed to rest on the HSVET chinrest and carry out the respective task for 15 min, until the examiner told them to stop. Sufficient material was provided for 15 min of reading without repetition.

During the last 150 s of the task (minutes 12.5–15), the subjects’ eye movements were recorded with the HSVET. Recordings were performed on the right eye in all subjects. Subjects were not actively told that their eyeblinks were being recorded. Each measurement generated a sequence of 37,500 images of the participants’ right eye that were stored onto an external hard drive and subsequently studied by means of image analysis, to obtain a non-invasive detailed description of the eye blink movement. The image processing–based method used for the automatic analysis of blinking has been previously described in the literature [17]. Blinks were evaluated in terms of kinematic variables including blink rate, number of complete and incomplete blinks, percentage of incomplete blinks, blink amplitude, opening and closing blink speeds, and opening, closing, contact, and total blink durations. A complete blink was defined as that in which the position of the superior eyelid reached the median height level of the inferior eyelid.

The experimental design and methodological procedure were similar to those used in previous studies [23].

### Statistical analysis

The results were evaluated using SPSS software v.26 (IBM Corp., Armonk, NY). The normality of data was assessed by using the Shapiro-Wilk test. When normality could be assumed, a repeated-measures ANOVA was used to examine the statistical significance of the blink kinematic variables for the 5 task conditions. The Mauchly test was used to evaluate the assumption of sphericity. If sphericity could not be assumed, the Greenhouse-Geisser correction was applied. Whenever the repeated-measures ANOVA pointed to a statistical significance, post hoc pairwise comparisons were carried out using the Bonferroni correction. The non-parametric Friedman test for repeated measures with the Dunn-Bonferroni post hoc analysis was used when parametric test assumptions were not fulfilled. *P*-values of < 0.05 were considered statistically significant. Estimated sample size was calculated a priori using the G-power tool, after a preliminary study in a small sample. The significance level was 5%, an allowance of 0.05 alpha error, and a study power of 80%. The calculated sample size was 29, after which 32 were finally recruited, allowing for possible study dropouts. Finally, the statistical power was calculated post hoc using the G-Power tool, being this superior to 0.83 (effect size = 0.53).

## Results

The sample of this study included 32 healthy young subjects ranging in age from 20 to 26 years (22.5 ± 1.6, mean ± SD). Table 1 presents the intra-average mean values and 95% confidence intervals of the blinking kinematic variables and characteristics assessed in the present study during the control measure and during the reading task with each device. The table also presents the statistical results of the comparison of all 5 examination conditions.

**Table 1:** Intra-average mean values and 95% confidence intervals of the blinking kinematic variables obtained during the control measure and during the reading task with each device and statistical results of the comparison

Variable	Control (CT)	Computer (C)	Tablet (T)	E-reader (Er)	Smartphone (S)	*P*-value	Statistically significant post hoc differences (*P*-value)
Blink rate (*total number of blinks*) (Blinks/min)	20.4 (*50.9*) [16.2 24.5]	9.8 (*24.6*) [6.9 12.8]	10.2 (*25.5*) [7.4 13.0]	9.7 (*24.2*) [7.4 12.0]	11.1 (*27.7*) [8.5 13.7]	*p* < 0.0005*	CT – C (*p* < 0.0005) CT – Er (*p* < 0.0005) CT – T (*p* < 0.0005) CT – S (*p* < 0.0005)
Number of complete blinks	33.5 [23.4 43.6]	11.5 [7.0 16.0]	15.1 [9.7 20.4]	15.2 [10.6 19.7]	25.7 [19.0 32.3]	*p* < 0.0005*	CT – C (*p* < 0.0005) CT – T (*p* < 0.0005) CT – Er (*p* < 0.0005) C – S (*p* < 0.0005) T – S (*p* = 0.034) Er – S (*p* = 0.038)
Number of incomplete blinks	17.4 [11.7 23.1]	13.1 [8.3 17.9]	10.5 [6.9 14.0]	9.1 [5.8 12.4]	2.1 [1.0 3.2]	*p* < 0.0005*	CT – Er (*p* = 0.006) CT – S (*p* < 0.0005) C – S (*p* < 0.0005) T – S (*p* < 0.0005) Er – S (*p* = 0.035)
Percentage of incomplete blinks (%)	37.9 [27.7 48.1]	56.0 [45.3 66.7]	42.8 [33.5 52.1]	37.4 [27.3 47.5]	10.1 [4.8 15.4]	*p* < 0.0005*	CT – C (*p* = 0.043) CT – S (*p* = 0.004) C – S (*p* < 0.005) C – Er (*p* = 0.030) T – S (*p* < 0.0005) Er – S (*p* = 0.015)
Amplitude (mm)	5.4 [4.8 6.0]	4.3 [4.0 4.7]	3.8 [3.5 4.1]	3.8 [3.5 4.2]	3.1 [2.7 3.4]	*p* < 0.0005*	CT – C (*p* = 0.003) CT – T (*p* < 0.0005) CT – Er (*p* < 0.0005) CT – S (*p* < 0.0005) C – T (*p* = 0.001) C – Er (*p* = 0.048) C – S (*p* < 0.0005) T – S (*p* = 0.001) Er – S (*p* < 0.0005)
Closing duration (ms)	41.6 [37.0 46.2]	38.9 [35.8 41.9]	42.1 [40.0 44.1]	43.0 [39.7 46.3]	40.4 [38.3 42.4]	*p* = 0.259	—
Contact duration (ms)	73.0 [59.3 86.6]	44.7 [39.3 50.2]	54.5 [46.4 62.5]	59.4 [49.8 69.0]	70.9 [58.4 83.3]	*p* = 0.001*	CT – C (*p* = 0.004) C – S (*p* = 0.001)
Opening duration (ms)	185.0 [156.7 213.3]	158.8 [133.5 184.1]	161.4 [134.8 188.0]	159.9 [135.8 184.1]	166.9 [147.0 186.8]	*p* = 0.148	—
Total duration (ms)	299.7 [261.2 338.1]	242.1 [214.7 269.5]	255.6 [226.9 284.3]	257.6 [232.0 283.3]	278.8 [255.0 302.6]	*p* = 0.009*	CT – C (*p* = 0.017)
Closing speed (mm/s)	139.2 [125.2 153.2]	118.6 [106.0 131.1]	96.0 [86.6. 105.3]	95.7[85.5 106.0]	76.4 [70.0 82.8]	*p* < 0.0005*	CT – S (*p* < 0.0005) CT – T (*p* < 0.0005) CT – Er (*p* < 0.0005) C – S (*p* < 0.0005) C – T (*p* = 0.007) C – Er (*p* = 0.023) T – S (*p* = 0.043) Er – S (*p* = 0.023)
Opening speed (mm/s)	39.8 [30.9 48.6]	37.5 [30.6 44.4]	30.7 [26.4 35.0]	30.6 [25.4 35.9]	21.5 [19.0 24.0]	*p* < 0.0005*	CT – S (*p* < 0.0005) C – S (*p* < 0.0005) T – S (*p* = 0.010) Er – S (*p* = 0.030)

^*^Indicates statistical significance

**Fig. 1:**
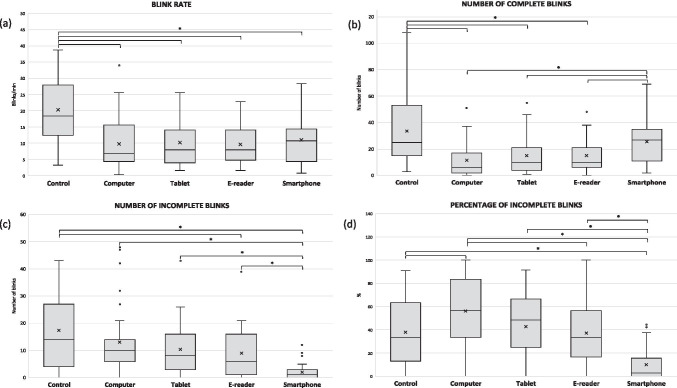
Boxplots of (**a**) blink rate, (**b**) number of complete blinks, (**c**) number of incomplete blinks, and (**d**) percentage of incomplete blinks obtained during the control task and during the reading task with each device. The asterisk indicates statistical significance

Figure 1 shows boxplots of the blink rate (a), number of complete blinks (b), number of incomplete blinks (c), and percentage of incomplete blinks (d) obtained in the control condition and during the reading task with each device. Post hoc comparisons revealed statistically significant differences in all variables amongst conditions (*p* < 0.0005 for all). Blink rate was significantly lower when reading on all digital displays compared to the control condition (*p* < 0.0005 for all), although no differences were obtained amongst devices (*p* > 0.05). The number of complete blinks performed was significantly lower when reading on all devices, except the smartphone, compared to the control task (*p* < 0.0005 for all). Likewise, the number of complete blinks was significantly higher when reading on the smartphone compared to the rest of the displays (*p* ≤ 0.038 for all).

Additionally, the number of incomplete blinks was significantly lower when reading on the smartphone in comparison to the rest of the devices (*p* ≤ 0.035 for all) and the control task (*p* < 0.0005) and was also lower when using the e-reader compared to the control (*p* = 0.006). Finally, the percentage of incomplete blinks was significantly lower when reading on the smartphone compared to the other 3 displays (*p* ≤ 0.015 for all) and the control condition (*p* = 0.004), while significantly more incomplete blinks were performed when reading on the computer in comparison to the control (*p* = 0.043) or when using the e-reader (*p* = 0.030).

**Fig. 2:**
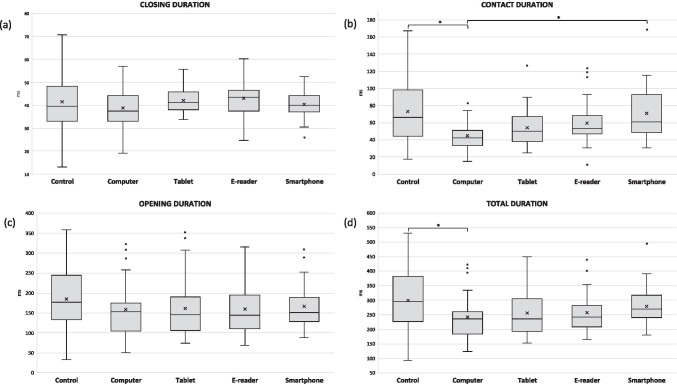
Boxplots of the duration of each of the phases of blinking obtained during the control task and during the reading task with each device. (**a**) Closing duration, (**b**) contact duration, (**c**) opening duration, and (**d**) total duration. The asterisk indicates statistical significance

Moreover, Fig. 2 shows boxplots of the duration of each of the phases of blinking for the 5 examination conditions. As evidenced, no differences in the closing (a) or opening (b) durations were obtained amongst conditions (*p* > 0.05). Nevertheless, blinks had a significantly lower contact time (c) when reading on the computer compared to the control measure (*p* = 0.004) and compared to reading on the smartphone (*p* = 0.001) and were overall (d) significantly shorter than in the control (*p* = 0.017).

**Fig. 3:**
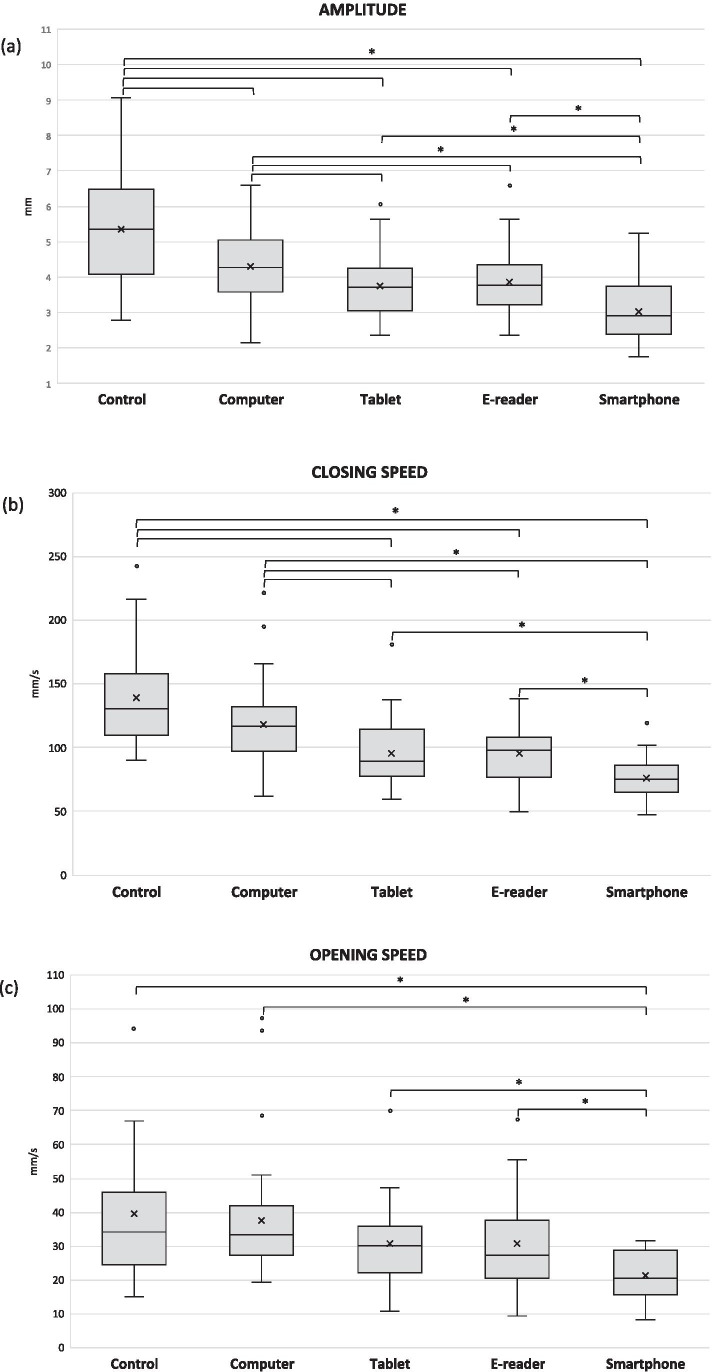
Boxplots of (**a**) the blinking amplitude and (**b**) the closing and (**c**) opening speeds obtained during the control task and during the reading task with each device. The asterisk indicates statistical significance

Finally, Fig. 3 shows the boxplots of the blinking amplitude (a) and the blinking closing (b) and opening (c) speeds. When reading on the smartphone, blinks had a significantly smaller amplitude in comparison to the other devices and the control measure (*p* ≤ 0.001 for all). On the contrary, blinks had a greater amplitude when reading on the computer compared to the tablet and the e-reader (*p* ≤ 0.048 for all) and when looking straight ahead in the control condition than during the digital display reading tasks (*p* ≤ 0.003 for all). Furthermore, blinks were significantly slower during the closing phase of blinking when using the smartphone in comparison to the rest of the conditions (*p* ≤ 0.043), while they were faster for the control and the computer compared to the tablet (*p* < 0.0005 and *p* = 0.007, respectively) and the e-reader (*p* < 0.0005 and *p* = 0.023, respectively). Lastly, the opening speed of blinking was significantly slower when reading on the smartphone compared to all the other digital displays (*p* ≤ 0.030) and the control measure (*p* < 0.0005).

## Discussion

The impact of digital display use on blinking is widely acknowledged and is globally accepted as the main mechanism leading to digital display–induced dry eye [3–10]. The process of blinking is key for preserving the ocular surface and tear film homeostasis, by maintaining adequate levels of humidity and hydration, promoting the expression of tear lipids and spreading them through the precorneal film, and helping in the drainage of tears, amongst other functions [24–26]. Blinking is a complex process, composed of different stages, each of which involves different muscle interactions [16]. Nevertheless, despite the complexity and relevance of blinking, no study up to date has addressed the influence of digital display use on blinking kinematics, with most studies focused merely on blink rate or number of incomplete blinks. Likewise, the appearance of handheld devices such as tablets, smartphones, or e-readers, which differ in many aspects of their pattern of use and characteristics, makes differences between them probable.

In the present study, the total number of blinks performed during the recording period, and consequently blink rate, was reduced by 45–55% when reading on all displays. As expected, this decrease in blink frequency was linked to a decrease in both the number of complete and incomplete blinks. This is in line with previous research and with the acknowledged mechanism behind ocular surface desiccation associated with computer usage [5–7, 27, 28]. Nevertheless, when it comes to handheld devices specifically, research is still limited.

Smaller screens lead to a lower amplitude of saccades and consequently no requirement of combined blinking, which has been suggested to reduce blink rate further [15, 29]. Nevertheless, in the present study, the text displayed on all devices was matched in many parameters, including page angular width and number of words per line and lines per page; thus, both the number of saccades and their amplitude remained constant when reading on all displays. Likewise, a correlation between gaze angle and blink frequency during computer use has been proposed. For instance, Nielsen et al. [14] found that lowering the position of the monitor decreased blink rate significantly. This decrease is suspected to be a direct consequence of the reduction in exposed ocular surface area. In the present study, handheld devices were associated with lower areas of ocular surface exposure attributed to closer distances of usage compared with the control and the computer: 154 ± 32 mm^2^ for the control, 140 ± 37 mm^2^ for the computer, 120 ± 29 mm^2^ for the tablet, 118 ± 42 mm^2^ for the e-reader, and 80 ± 31 mm^2^ for the smartphone. However, blink rate remained constant amongst displays with differences being both statistically and clinically negligible.

As previously mentioned, the e-reader is based on E-ink technology which simulates printed paper. Considering both modes of presentation (i.e., e-reader and printed paper) to be equivalent, blink rate was probably controlled by the cognitive demand of the task rather than the form of presentation. Therefore, the marked reduction in blink rate when using the displays was probably attributed to the increased cognitive demand of the reading task [3, 8], in comparison to the low cognitive demand of the control measure, and not to the nature of the displays. After comparing the blink rate of 25 subjects who performed a 20-min reading task on either a desktop computer screen or a printed hard copy page with matched characteristics, Chu et al. [9] concluded that “previously observed differences in blink rate were more likely to be produced by changes in cognitive demand rather than the method of presentation,” Later, Rosenfield et al. [30] confirmed this hypothesis and pointed to incomplete blinking as the current cause behind the dryness symptoms experienced by users with modern digital displays.

As for incomplete blinking, more than half of the blinks performed during computer use were incomplete, this number being on average considerably higher when compared to the control task (56.0% vs 37.9%, respectively). Conversely, the number of incomplete blinks was greater during the control task than during computer and e-reader usage, although this was probably a direct consequence of the significantly lower blink rate obtained while reading. This is in line with previous research and explains the evoked dry eye signs and symptoms during computer operation [3, 4, 9, 30]. Harrison et al. [31] pointed out that incomplete blinks may occur to not interrupt concentration, which links to the suggestion that incomplete blinks may be the result of unsuccessful inhibition of a spontaneous blink during visually demanding tasks [32].

Interestingly, both the proportion and the number of incomplete blinks gradually decreased as the displays were positioned closer and at lower gaze angles, reaching statistical significance when using the smartphone, in comparison to the other devices and even the control measurement. To the authors’ knowledge, no study to date has directly addressed the relationship between gaze angle or distance and incomplete blinking. A simple explanation for the decrease in incomplete blinking from upward to downward gaze may be that with smaller palpebral fissures the distance the upper eyelid must travel is shorter, increasing the chances of coming into contact with the lower eyelid margin. Contrary to our results, Golebiowski et al. [33] found an increase in incomplete blinks with the duration of smartphone visualization. Similarly, Argilés et al. [15] obtained a greater percentage of incomplete blinks while reading on a tablet (14.5%) compared to printed text (5%). Nevertheless, the lack of studies involving handheld devices, along with the differences in experimental conditions and settings, makes comparisons challenging.

According to our outcomes and to a greater ocular surface exposure, the computer may have the biggest impact on the ocular surface and the tear film, while the smartphone may partially prevent ocular dryness. Novel research comparing the effects of reading on several digital displays revealed that reading on a laptop computer for a brief period leads to greater dry eye symptoms, lower tear volume and tear stability, and higher osmolarity and conjunctival redness compared to reading on handheld devices or a non-device baseline measurement [23].

No studies published to date have examined the impact of gaze angle on blink amplitude with digital devices including computers. In the present study, blink amplitude was greater during the control task (i.e., looking at a fixation target at eye level) and decreased significantly as the angle of usage of the displays decreased, probably due to the close relationship between gaze angle and palpebral fissure. Despite the difference in blink amplitude, closing and opening blink durations remained unvaried amongst conditions and therefore, closing and opening speeds were progressively slower, with blinks being significantly slower when reading on the smartphone and faster when reading on the computer or during the control task. The relationship between the amplitude of a blink and its maximum speed is considered to be linear [16, 17, 34]. This characteristic of blinking is known as the main sequence and indicates that as the amplitude increases so does maximum speed, which means that the greater the distance that a blink covers, the faster it is [11, 35].

In addition to this, contact duration was slower while reading on the computer than during the control task or when reading on the smartphone, which consequently resulted in a shorter overall blink duration compared to the control task. This shorter contact duration was probably attributed to a significantly higher percentage of incomplete blinks during computer usage. A blackout in the visual input to the brain occurs each time we blink. However, this periodic decrement in retinal luminance is not perceived due to blink suppression, in which neural activity involved in visual perception is actively reduced during blinking [36]. This suppression occurs not only during blinking but also 50 to 100 ms and 100 to 150 ms before and after a blink, respectively, with the total time lost being dependent on the duration of the blink [36]. Given the greater amplitude of blinks with higher palpebral fissures, along with the cognitive-demanding reading task, a higher proportion of incomplete blinks may have been unconsciously performed during computer reading in an attempt to minimize the duration of the contact phase of blinking and the associated blackout in the visual input.

Finally, the present study had some limitations to consider. Given the high temporal resolution of the image recording device (250 Hz), the duration of blink recording was limited by data volume and chosen as a compromise between sampling time and volume of information. Also, the results had some limitations attributable to the image processing technique used, which have already been described in detail elsewhere [17]. Finally, given the lack of studies assessing blinking kinematics during screen use, some of the results could not be contrasted with the literature and, therefore, further studies are required to confirm these findings.

In conclusion, blink rate was significantly reduced when reading on all displays compared to a non-device low demanding control task, probably as a consequence of the higher cognitive demand of reading, while no differences amongst digital displays were obtained. Incomplete blinking increased as displays were positioned further and at greater gaze angles, being greater while reading on the computer, possibly due to a summative effect between a larger palpebral fissure and a higher cognitive demand. Blink amplitude was directly related to gaze angle, being lower for devices with associated smaller visualization angles and higher for displays with greater angles of usage. Furthermore, closing and opening blink durations remained unvaried amongst displays while opening and closing speeds were greater for the computer and the control measurement and reduced progressively with gaze angle and distance, being the lowest for the smartphone. Finally, total blink duration was shorter during computer operation compared to the control, probably because of a longer contact duration associated with a higher percentage of incomplete blinks.

Overall, the present study highlights the relevance of fully characterizing the process of blinking during digital display use and provides the basis for future studies in this field. Furthermore, the present study underlines the utility of image processing–based methods using high-speed video cameras to precisely and non-invasively analyze blink kinematics while using digital displays.
